# An Inhibitive Enzyme Assay to Detect Mercury and Zinc Using Protease from *Coriandrum sativum*

**DOI:** 10.1155/2013/678356

**Published:** 2013-09-30

**Authors:** Gunasekaran Baskaran, Noor Azlina Masdor, Mohd Arif Syed, Mohd Yunus Shukor

**Affiliations:** ^1^Department of Biochemistry, Faculty of Biotechnology and Biomolecular Sciences, Universiti Putra Malaysia, 43400 Serdang, Selangor, Malaysia; ^2^Biotechnology Research Centre, MARDI, P.O. Box 12301, 50774 Kuala Lumpur, Malaysia

## Abstract

Heavy metals pollution has become a great threat to the world. Since instrumental methods are expensive and need skilled technician, a simple and fast method is needed to determine the presence of heavy metals in the environment. In this study, an inhibitive enzyme assay for heavy metals has been developed using crude proteases from *Coriandrum sativum*. In this assay, casein was used as a substrate and Coomassie dye was used to denote the completion of casein hydrolysis. In the absence of inhibitors, casein was hydrolysed and the solution became brown, while in the presence of metal ions such as Hg^2+^ and Zn^2+^, the hydrolysis of casein was inhibited and the solution remained blue. Both Hg^2+^ and Zn^2+^ exhibited one-phase binding curve with IC_50_ values of 3.217 mg/L and 0.727 mg/L, respectively. The limits of detection (LOD) and limits of quantitation (LOQ) for Hg were 0.241 and 0.802 mg/L, respectively, while the LOD and LOQ for Zn were 0.228 and 0.761 mg/L, respectively. The enzyme exhibited broad pH ranges for activity. The crude proteases extracted from *Coriandrum sativum* showed good potential for the development of a rapid, sensitive, and economic inhibitive assay for the biomonitoring of Hg^2+^ and Zn^2+^ in the aquatic environments.

## 1. Introduction

Human activity in the last few decades has led to global contamination by organic and inorganic compounds [1, 2]. The presence of the pollutants generated from industrial and agriculture activities in the waterways has been identified to produce potential harmful effect on the aquatic living organisms and the food webs [3–6]. Nowadays, heavy metal contamination is considered to be among the most serious environmental problems. Heavy metals are any inorganic metallic compounds that can exert their toxicity via binding to the thiol group and disulfide bond that contribute to the stability of the enzyme [7]. The metals have high affinity to the disulfide bridge between two cysteine residues in any protein compound. Heavy metals are very dangerous to living organisms especially for humans since they can cause DNA damage and exert carcinogenic effects. In Malaysia, Juru Industrial Estate is renowned for releasing elevated concentrations of heavy metals into agricultural and aquaculture areas [8]. In addition, Alina et al. [9] reported that fishes in the coastal areas of Malaysia were contaminated by heavy metals. Hence, it is essential to monitor heavy metals in Juru area. The use of classical methods such as atomic absorption spectroscopy is expensive, requires highly-trained operators, complicated sample pretreatment, and needs a long measuring period [8]. Therefore, simple and fast techniques are really needed for the detection of heavy metals in the environment.

Inhibitive enzyme assays have been long developed to detect toxicants like heavy metals because of their rapid and economic approach. Recent works on the development of inhibitive enzyme assays involved the use of proteases such as papain, bromelain, and trypsin to detect heavy metals [8, 10, 11]. In general, bioassay is nonspecific towards a particular heavy metal, but it can be used as an early monitoring system [12, 13]. In this study, a novel source of protease from a local plant for detection of mercury (Hg) and zinc (Zn) was investigated.

## 2. Materials and Methods

### 2.1. Preparation of Buffer Solutions

The buffers were prepared according to the methods of  Dawson et al. [14]. Adjustment to the pH of buffer was made using NaOH (5 N) and HCl (5 N).

### 2.2. Bradford Dye Binding Assay

Bradford reagent was prepared by mixing Coomassie Brilliant Blue G-250 (100 mg; Sigma, St. Louis, USA) with 95% ethanol (50 mL) and 85% phosphoric acid (100 mL) [15]. The solution was made up to 1 L and stirred overnight. The solution was filtered through filter paper (Whatman Filter Paper No. 1, GE Healthcare, Pittsburgh, PA, USA) and stored in dark bottles [16, 17].

### 2.3. Preparation of Casein Solution

Casein was prepared according to the method of Shukor et al. [11]. Casein (2 g; Sigma, St. Louis, USA) was dissolved in deionised water (100 mL) and the pH was adjusted to 8.0. The casein stock solution was then incubated overnight at 60°C under a mild stirring condition. The solution was filtered through several layers of cheesecloth [18], and the filtrate was centrifuged at 10,000 ×g for 10 min. The protein concentration of casein in the supernatant was measured by Bradford dye-binding assay using BSA (Sigma, St. Louis, USA) as a standard.

### 2.4. Preparation of Heavy Metals Solutions

Silver, arsenic, cadmium, cobalt, chromium, copper, mercury, nickel, lead, and zinc stock solutions were purchased from Merck, Darmstadt, Germany. The working solutions (10 mg/L, 5 mg/L, 2.5 mg/L, 1.0 mg/L, and 0.5 mg/L) were prepared using deionized water and stored in acid-washed polypropylene containers.

### 2.5. Extraction of Plant Protease

Extraction of plant proteases was carried out according to the modified method of Jiang et al. [19]. Chopped plant tissues were immersed in sodium phosphate buffer (50 mM; pH 7) for 2 days in the chiller. Homogenization buffer was added (1 ratio of plant : 3 ratios of chilled buffer) and the mixture was blended for 20 s at high speed followed by a 10 min of cooling period. The cycle of blending and cooling was repeated until plant samples were homogenized. The products were sieved and centrifuged at 10,000 ×g for 15 min at 4°C. The pellet and supernatant were further assayed to determine proteases activity. 

### 2.6. Optimization of Enzyme Assay

The optimization of enzyme concentrations, substrate concentrations, pH, temperature, and incubation time was employed to obtain an optimum absorbance for the detection of heavy metals. The absorbance range between 0.3 and 0.9 is the ideal difference from the blank to ensure visible color changes [8]. This is important for qualitative detection of heavy metals since it is not possible to bring spectrophotometer to the field.

### 2.7. Protease Inhibition Studies

Protease inhibitive assay for heavy metals was performed as described by Shukor et al. [8]. Protective reagents like EDTA and DTT were removed to enhance the sensitivity towards heavy metals [7]. Protease (100 *μ*L) was added to sodium carbonate buffer (20 *μ*L; 50 mM; pH 9.0) in a microcentrifuge tube followed by the addition of heavy metals (20 *μ*L). For the control, deionized water (20 *μ*L) was used instead of heavy metals. Casein (60 *μ*L) was added to the mixture after 20 min of incubation at room temperature. An aliquot (20 *μ*L) was withdrawn and mixed with Bradford dye-binding reagent (200 *μ*L) in a microtiter plate well. The mixture was incubated for 5 min and the absorbance was designated as time zero. The remaining solution was incubated at 35°C for 20 min. After the incubation period, another aliquot (20 *μ*L) was collected and treated in the same manner as the aliquot at time zero. The absorbance at 595 nm was measured using a microtiter plate reader (Stat Fax 3200 Microplate Reader, Awareness Technology Inc., USA). The values for the IC_50_ (inhibitory concentration, 50%) were calculated using the nonlinear regression analysis for one-phase binding model using the GraphPad PRISM 5 Software. Means and standard deviation were determined based on three independent experimental replicates.

### 2.8. Collecting of Environmental Samples (Field Trials)

Samples were collected from aquatic environments from several industrial outlets that release heavy metals products such as pristine areas and galvanized metals factories. In this study, 2 states in Malaysia were targeted for the sampling works: Prai and Bukit Tengah Industrial Areas (Penang) and Endau Rompin National Park (Johor). Water samples were taken approximately 20–30 cm from the surface of the water. The collected samples were placed in the acid-washed HDPE bottles. Several drops of concentrated nitric acid (HNO_3_) were added to extract heavy metals that bound to other compounds in the samples. The samples were filtered by using 0.45 *μ*m filter membrane and finally assayed in inhibitive protease assay and analyzed by ICP-OES.

## 3. Results and Discussion

### 3.1. Screening towards Plant Protease

Identification of the plant source that gives the highest enzyme activity is very crucial in this study since bioassay is only practical for enzyme that shows high enzymatic activity. The significant color changes of the Bradford reagent are only observable if the protease has high activity.  Table 1 shows the percentage activity between plant sources. Commercialized enzyme, papain (Sigma, E.C. 3.4.22.2, lot no: 32K2619, St. Louis, USA), was used as a positive control, and enzyme was replaced with deionized water for the negative control.

Among all plant samples, *Coriandrum sativum* gave the highest enzyme activity. Although high enzyme activity does not correlate with the sensitivity of the enzyme towards heavy metals, this plant source was used since the activity is sufficiently high for inhibitive assay. The inhibitive assay is only valid if the difference in absorbance is more than 0.2 after a maximum incubation for 1 h [8]. *Coriandrum sativum *has senescence-related serinyl protease which is involved in the physiological role and metabolic activities of the plant [19]. 

### 3.2. Optimized Conditions for* Coriandrum sativum* Protease Activity

The optimum concentration of *Coriandrum sativum* proteases was at 45 *μ*g/mL. The optimum concentration was higher compared to previous optimization studies on other proteases such as papain, bromelain, and trypsin as shown in Table 2. The optimized concentration would ensure that degradation was complete before any autodigestion of proteases takes place. Substrate casein gave optimum activity at 42.5 *μ*g/mL. Higher concentration of the substrate is not desired since it would take a longer time for the casein to be digested. The optimized temperature was at 35°C, lower compared to the proteases such as bromelain and trypsin [10, 11]. Thus, it does not require extra heating process since it is almost similar to the environmental temperature in Malaysia. The highest enzyme activity was noted at incubation period of 20 min and at the pH range between 8 and 9.5 in sodium carbonate buffer. The broad range of pH is favored for assaying environmental samples since it can bear deviation in samples' pH [11].

### 3.3. Heavy Metals Studies

**Figure 1:**
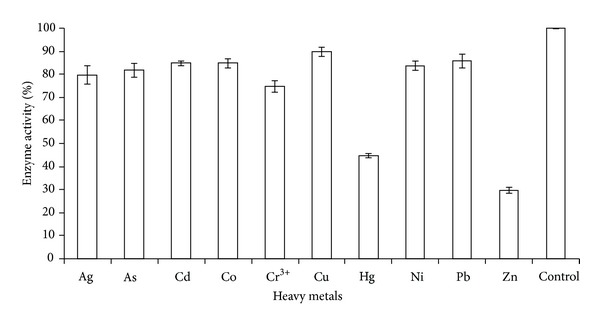
The effect of heavy metals at 1 mg/L on *Coriandrum sativum* proteases activity. All data are expressed as mean ± SEM.

The *Coriandrum sativum* protease activity was inhibited by two heavy metals: mercury and zinc, at 1 mg/L as shown in Figure 1. The other heavy metals did not show significant inhibition towards the enzyme activity; thus, the solution remained brown in color. This is because Bradford reagent is unable to stain digested casein which is less than 2 kDa [10]. Mercury and zinc were observed to exhibit dark blue color after the completion of the assay indicating that both heavy metals inhibit the activity of the protease. The mechanism of metal inhibition on enzyme activity especially by mercury is through binding of the sulfhydryl groups in cysteine proteases and through destruction of tertiary structure in which the disulfide bridges were destroyed and the casein became unfit to the active site of the enzyme [8]. Therefore, Bradford reagent stains the undigested casein and the solution changed to dark blue in color. The *Coriandrum sativum* proteases probably contain a mixture of serine [19] and cysteine proteases. Serine proteases are known to be inhibited by zinc [11], while cysteine proteases are strongly inhibited by mercury [8]. Therefore, the inhibitive enzyme assay using *Coriandrum sativum* is a potential candidate for biomonitoring of Hg and Zn in aquatic environments.

### 3.4. Comparison of the Half Maximal Inhibitory Concentration (IC_50_) Values of Proteases

**Figure 2:**
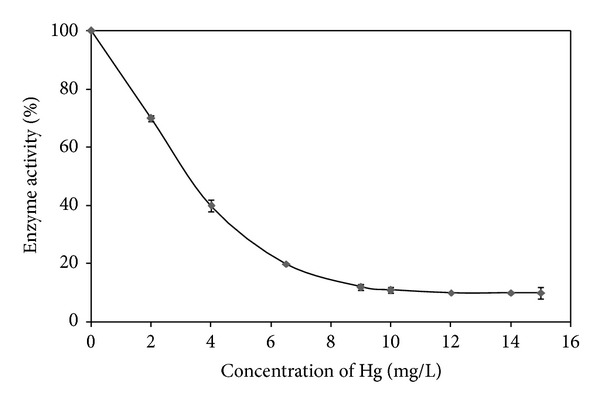
in which the disulfide bridges were destroyed and the casein became unfit to the active site of the enzymeInhibition of proteolytic activity of *Coriandrum sativum* protease by Hg using Coomassie brilliant blue assay. Data is generated using the nonlinear regression analysis for one-phase binding model using the GraphPad PRISM 5 Software. All data are expressed as mean ± SEM.

**Figure 3:**
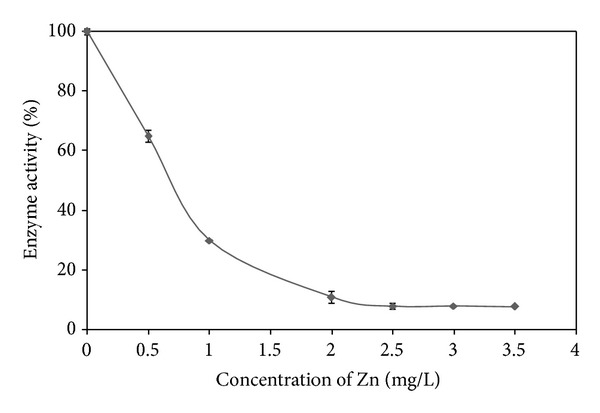
Inhibition of proteolytic activity of *Coriandrum sativum* protease by Zn using Coomassie brilliant blue assay. Data is generated using the nonlinear regression analysis for one-phase binding model using the GraphPad PRISM 5 Software. All data are expressed as mean ± SEM.

In terms of sensitivity, the IC_50_ values are often used as a comparison among established assays [4]. The comparison of IC_50_ values of proteases studied so far with other types of assays is summarized in Table 3. For Hg, *Coriandrum sativum* proteases showed better sensitivity compared to trypsin with the IC_50_ value of 3.217 mg/L (Figure 2). Meanwhile, the IC_50_ value for Zn is 0.727 mg/L (Figure 3), which is more sensitive than other assays like papain, bromelain, trypsin, and immobilized urease, and within the range of Microtox and *Daphnia magna* assays. The lower IC_50_ value denotes the higher effectiveness of protease in inhibiting heavy metals. The advantages of inhibitive assay in comparison with Microtox are that it is inexpensive and suitable for the detection of heavy metals in field trials since it is based on colorimetric changes which do not require photometer. Meanwhile, *Daphnia magna* assays have broad range of IC_50_ value and need a longer period of incubation, 48 h compared to inhibitive assay which only requires 20 min.

### 3.5. Limits of Detection (LOD) and Limit of Quantitation (LOQ) for Hg and Zn

The LOD and LOQ values for Hg and Zn detected by coriander proteases are shown in Table 4. LOD is the lowest concentration of heavy metal that can inhibit the enzyme activity, which is at least three times the standard deviation of the blank at *y*-intercept [20]. The LOD values of Hg and Zn are 0.241 and 0.228 mg/L, respectively. Meanwhile, LOQ indicates the minimal concentration of heavy metal that can produce observable color changes to the Bradford assay. LOQ is ten times the value of the standard deviation of the mean blank value [20]. The LOQ values of Hg and Zn are 0.802 and 0.761 mg/L, respectively. The LOQ value for Hg was lower and about the same for Zn compared to the trypsin assay, 1.35 and 0.61 mg/L, respectively, [11]. Thus, proteases from this plant have a promising potential to be used as an inhibitive assay for Hg and Zn.

### 3.6. Field Trials

Samples were collected from Prai and Bukit Tengah Industrial Areas and Endau Rompin National Park. The samples were analyzed using the inhibitive assay and the results were validated with ICP-OES. Twelve out of 24 samples from Prai Industrial Area gave more than 50% inhibition to the protease activity (Table 5). ICP-OES results showed that the positive samples contain extremely high concentration of Hg and Zn with some exceeding the maximum permissible limit (MPL) for Hg and Zn, 0.001 and 5 mg/L, respectively, as outlined by the Department of Environment (DOE) [21]. LOQ value for Hg, 0.802 mg/L, is much higher than the MPL for Hg in aquatic environment as outlined by the DOE of Malaysia, 0.001 mg/L. However, this assay can be used in monitoring Hg levels in industrial sites since these areas mostly contain very high concentration of Hg. Previous studies carried out in the same area also reported that the area is highly polluted with heavy metals [8, 10], indicating a continuous trend.

Four out of 5 samples from Bukit Tengah Industrial Area (Table 6) gave positive inhibitory result on enzyme activity due to the presence of Hg and Zn as determined by ICP-OES. Since galvanized metal factories can be found in this area, it is suspected that these types of industries are responsible for the elevated Hg and Zn level. Thus, a fast and inexpensive method for monitoring these heavy metals is urgently needed.

Endau Rompin is the one of the largest national park in Malaysia with pristine water and a diverse mixture of flora and fauna. Four distinct samples were taken from this area and the results showed that there was no significant inhibitory effect on enzyme activity. The results obtained from ICP-OES also proved the absence of heavy metals (Table 7) indicating a good correlation between inhibitive assay and instrumental method.

## 4. Conclusion

An assay for detecting Hg and Zn using proteases from a local plant has been successfully developed. The optimizations of enzyme and substrate concentration, pH, temperature and incubation time were performed using the Bradford-protease-casein system. In field trials, samples obtained from polluted and nonpolluted sites showed promising results. The findings showed that Prai and Juru Industrial Areas are highly polluted with Hg and Zn. The protease has a broad pH range and high sensitivity towards Hg and Zn suggesting robustness and suitability for biomonitoring field works. For future studies, it is recommended that the proteases be purified to increase the sensitivity towards the heavy metals. This study provides fundamental information for the development of rapid, sensitive, and economic inhibitive assay for the biomonitoring of heavy metals in the environment.

## Figures and Tables

**Table 1:** Enzyme activity of different plant crude extracts.

Samples	Percentage of activity (%)
Negative control	0 ± 0.5
*Averrhoa carambola* (Star fruit)	10 ± 1.0
*Ipomoea batatas* (Sweet potato)	12 ± 0.5
*Solanum melongena* (Brinjal)	15 ± 1.5
*Mangifera similis* (Mango)	14 ± 0.5
*Cucumis sativus* (Cucumber)	49 ± 1.0
*Coriandrum sativum* (Coriander)	95 ± 1.0
*Citrus aurantiifolia* (Lime)	21 ± 0.5
*Murraya koenigii* (Curry leaf)	32 ± 1.0
Positive control	100 ± 0

All data are expressed as mean ± SEM.

**Table 2:** Summary of optimization results for *Coriandrum sativum* proteases in comparison to other proteases.

Optimization parameter	Optimum conditions for proteases			
	*Coriandrum sativum *	Papain^a^	Bromelain^b^	Trypsin^c^
Enzyme concentration (mg/mL) Substrate concentration (mg/mL) Temperature (°C) Incubation time (min) pH	0.45 0.425 35 20 8–9.5	0.1 0.1 30 30 5–7	0.1 0.25 40 30 5-6	0.1 0.1 40 30 5–7

^a^Shukor et al. [8].

^b^Shukor et al. [10].

^c^Shukor et al. [11].

**Table 3:** IC_50_ values of *Coriandrum sativum* proteases for Hg and Zn in comparison to other protease assays.

IC_50_ (mg/L)							
Heavy metals	*Coriandrum sativum *proteases	Papain^a^	Bromelain^b^	Trypsin^c^	Immobilized Urease^d^	15 min Microtox^d^	48-hour *Daphnia magna* ^d^
Hg	3.217	0.24–0.62	0.13–0.16	15.76–17.04	0.33	0.029–0.05	0.005–0.21
Zn	0.727	2.11	—	4.8–6.7	14.6	0.27–29	0.54–5.1

^a^Shukor et al. [8].

^b^Shukor et al. [10].

^c^Shukor et al. [11].

^d^Jung et al. [13].

**Table 4:** LOD and LOQ values for the proteases from *Coriandrum sativum. *

Metals	Regression model	R^2^	LOD (mg/L)	LOQ (mg/L)
Hg	One-phase binding	0.966	0.241	0.802
Zn	One-phase binding	0.984	0.228	0.650

**Table 5:** Protease inhibitive assay for samples from Prai Industrial Area.

Sample	GPS location	Percentage activity (%)	Concentration of heavy metals in samples (mg/L) by ICP-OES	
			Zn	Hg
1	N05°20.87′ E100°24.692′	93.59 ± 0.1	0.04 ± 0.05	n.d.
2	N05°20.87′ E100°24.692′	1.34 ± 0.3	125.30 ± 0.33	n.d.
3	N05°20.862′E100°24.674′′	98.54 ± 0.2	0.21 ± 0.12	n.d.
4	N05°20.836′ E100°25.177′	22.54 ± 0.5	7.63 ± 0.05 − 9	n.d.
5	N05°20.224′ E100°26.302′	98.80 ± 0.7	0.06 ± 0.03	n.d.
6	N05°21.983′ E100°24.023′	38.80 ± 0.9	0.89 ± 0.01	n.d.
7	N05°21.967′ E100°24.044′	93.98 ± 0.1	0.14 ± 0.02	n.d.
8	N05°20.87′ E100°24.692′	9.46 ± 0.1	14.54 ± 0.13	n.d.
9	N05°20.87′ E100°24.692′	25.21 ± 0.8	3.97 ± 0.04	n.d.
10	N05°20.862′ E100°24.674′	33.66 ± 0.2	3.50 ± 0.02	n.d.
11	N05°19.699′ E100°26.129′	19.21 ± 0.3	8.74 ± 0.09	n.d.
12	N05°19.699′ E100°26.129′	6.82 ± 0.3	12.00 ± 0.02	1.52 ± 0.06
13	N05°19.699′ E100°26.129′	8.77 ± 0.8	15.80 ± 0.13	n.d.
14	N05°20.263′ E100°25.774′	99.94 ± 0.9	0.02 ± 0.01	n.d.
15	N05°20.263′ E100°25.774′	99.39 ± 0.4	0.03 ± 0.03	n.d.
16	N05°20.135′ E100°26.925′	3.39 ± 0.3	38.65 ± 0.18	3.74 ± 0.09
17	N05°21.153′ E100°26.073′	94.81 ± 0.5	0.02 ± 0.02	n.d.
18	N05°21.153′ E100°26.073′	97.42 ± 0.7	0.09 ± 0.01	n.d.
19	N05°21.153′ E100°26.073′	98.11 ± 0.1	0.02 ± 0.01	n.d.
20	N05°20.091′ E100°25.269′	97.46 ± 0.2	0.06 ± 0.02	n.d.
21	N05°21.132′ E100°25.081′	99.24 ± 0.2	0.71 ± 0.01	n.d.
22	N05°20.472′ E100°26.891′	16.05 ± 0.3	10.84 ± 0.06	n.d.
23	N05°20.387′ E100°24.429′	4.95 ± 0.4	0.05 ± 0.01	n.d.
24	N05°20.263′ E100°25.774′	2.09 ± 0.7	12.69 ± 0.03	4.14 ± 0.15

All data are expressed as mean ± SEM.

n.d.: not detected.

**Table 6:** Protease inhibitive assay for samples from Bukit Tengah Industrial Area.

Sample	GPS location	Percentage activity (%)	Concentration of heavy metals in samples (mg/L) by ICP-OES	
			Zn	Hg
1	N05°20.447′E100°26.403′	6.39 ± 0.4	18.65 ± 0.03	n.d.
2	N05°20.665′ E100°26.364′	96.84 ± 0.7	n.d	n.d.
3	N05°20.601′ E100°26.427′	3.87 ± 0.5	13.42 ± 0.01	n.d.
4	N05°20.640′ E100°26.470′	32.47 ± 0.9	n.d.	2.36 ± 0.01
5	N05°18.947′ E100°26.348′	48.70 ± 1.0	n.d.	1.57 ± 0.02

All data are expressed as mean ± SEM.

n.d.: not detected.

**Table 7:** Protease inhibitive assay for samples from Endau Rompin.

Samples	GPS location	Percentage activity (%)	Concentration of heavy metals in samples (mg/L) by ICP-OES	
			Zn	Hg
1	N02°30.674′ E103°21.387′	97.49 ± 0.3	n.d.	n.d.
2	N02°30.802′ E103°21.086′	97.68 ± 0.2	n.d.	n.d.
3	N02°30.783′ E103°21.140′	95.93 ± 0.5	n.d.	n.d.
4	N02°30.784′ E 103°21.02′	99.53 ± 0.7	n.d.	n.d.

All data are expressed as mean ± SEM.

n.d.: not detected.
